# Characterization of the Gut Microbiota in Patients with Psoriasis: A Systematic Review

**DOI:** 10.3390/pathogens14040358

**Published:** 2025-04-07

**Authors:** Yingjun Gao, Yanfeng Lou, Yun Hui, Huan Chen, Hong Sang, Fang Liu

**Affiliations:** 1 Department of Dermatology, Jinling Hospital, Nanjing Medical University, Nanjing 210002, China; 2 Department of Stomatology, Jinling Hospital, Affiliated Hospital of Medical School, Nanjing University, Nanjing 210002, China; 3 Department of Dermatology, Jinling Hospital, Affiliated Hospital of Medical School, Nanjing University, Nanjing 210002, China

**Keywords:** psoriasis, gut microbiome, probiotics, fecal microbiota transplantation

## Abstract

**Background:** Psoriasis is a prevalent and persistent inflammatory disorder with systemic manifestations. Emerging evidence implicates the gut microbiota in regulating inflammatory responses, metabolic pathways, and immune homeostasis. This review synthesizes current evidence on gut microbiota dysbiosis in psoriasis and evaluates the therapeutic potential of probiotics and fecal microbiota transplantation (FMT) in disease management. **Method:** Following PRISMA guidelines, we systematically reviewed studies investigating gut microbiome profiles in psoriasis through the MEDLINE, EMBASE, and Web of Science databases (January 2015–December 2024). Included studies utilized 16S rRNA gene sequencing or metagenomic analyses for microbial characterization. **Results:** Comparative analyses revealed distinct gut microbiota patterns in psoriasis patients compared with healthy controls, although specific microbial signatures exhibited inconsistencies across studies. Notably, interventions modulating gut microbiota composition—particularly probiotic supplementation—demonstrated measurable improvements in psoriasis severity scores and inflammatory markers. **Conclusions:** Gut microbiome modulation represents a promising therapeutic strategy for psoriasis; however, current evidence highlights the need for standardized microbial analysis methodologies and larger longitudinal studies to establish causality. Future research should prioritize the functional characterization of microbiota–host interactions to optimize therapeutic applications.

## 1. Introduction

Psoriasis is a chronic, immune-mediated, inflammatory disease characterized by dysregulation of the tumor necrosis factor (TNF)-α/interleukin (IL)-23/IL-17 axis, affecting approximately 2–4% of the global population [1]. This systemic inflammatory disorder is strongly associated with comorbidities, including inflammatory bowel disease (IBD), metabolic syndrome, cardiovascular disease, and depression. Notably, psoriasis patients exhibit a 2.5-fold increased risk of Crohn’s disease (CD) and a 1.7-fold elevated risk of ulcerative colitis (UC), suggesting bidirectional crosstalk between systemic inflammation and gut mucosal immunity [2,3].

Emerging evidence highlights the dual role of the gut microbiota in psoriasis pathogenesis. While systemic inflammation may induce gut dysbiosis, impaired immune tolerance to commensal microbiota conversely exacerbates psoriatic flares. Clinically, CD patients exhibit a five-fold higher incidence of psoriasis [3], whereas murine studies demonstrated that antibiotic-induced gut microbiota modulation attenuates imiquimod (IMQ)-driven skin inflammation via suppressing Th17 responses [4]. Mechanistically, Chen et al. [5] identified that *Lacticaseibacillus pentosus* GMNL-77 ameliorates psoriatic lesions through downregulation of splenic Th17/Th22 cells and associated inflammatory cytokines. Similarly, Stehlikova et al. [6] reported that broad-spectrum antibiotics or metronidazole monotherapy reduced IMQ-induced skin pathology through Th17 pathway inhibition.

These findings collectively establish the gut–skin axis as a pivotal therapeutic target. Elucidating microbiota–host interactions could facilitate novel microbiome-directed therapies, including probiotics, antibiotics, or fecal microbiota transplantation (FMT), to concurrently mitigate both cutaneous and systemic inflammation in psoriasis.

## 2. Methods and Materials

This review was performed following the PRISMA guideline. We conducted a systematic literature search across the PubMed, Embase, and Web of Science databases using predefined Medical Subject Headings (MeSH) terms, which are summarized in Table 1. The search encompassed publications from January 2015 to December 2024, supplemented by manual screening of references in retrieved articles to minimize selection bias. To ensure methodological rigor, the PRISMA checklist was applied for quality assessment, with a PRISMA flow diagram outlining study inclusion/exclusion criteria. In accordance with the revised taxonomic classification of the genus *Lactobacillus* by Zheng et al. (2020) [7], wherein original *Lactobacillus* strains were reclassified into 25 distinct genera, all bacterial strains in this study have been updated to their latest taxonomic nomenclature.

### 2.1. Inclusion/Exclusion Criteria

Inclusion and exclusion criteria for studies investigating gut microbiota–psoriasis interactions, probiotic interventions, or FMT in psoriasis patients are detailed in Table 1. All identified records underwent dual independent screening against predefined eligibility criteria: (1) observational studies (case–control or cohort designs) examining human associations, mandating culture-independent genomic methods (e.g., 16S rRNA gene sequencing or shotgun metagenomics) for microbial profiling (or eligible interventional studies evaluating probiotics or FMT trials in psoriasis patients); (2) English-language full-text availability. We excluded (1) preclinical models (animal studies) and non-original research (reviews, conference abstracts, case reports); (2) studies lacking healthy control groups or employing culture-dependent microbial analysis; (3) opinion-based publications (editorials, expert commentaries). Additionally, studies were excluded if participants had comorbidities when assessing probiotics or FMT for psoriasis treatment.

### 2.2. Study Selection

Two investigators (Y. Gao and F. Liu) independently performed title/abstract screening of all retrieved publications, using predefined eligibility criteria to minimize selection bias. Articles identified as potentially relevant underwent full-text review to evaluate their alignment with inclusion/exclusion criteria. A senior researcher (Y. Hui) provided methodological supervision and arbitrated discrepancies through consensus-building discussions to ensure strict compliance with PRISMA guidelines.

### 2.3. Data Extraction

Two investigators (F. Liu and H. Sang), in a pair-wise manner, extracted the following data from the eligible studies: first author, publication year, participant information (sample size, age, sex), microbial profiling (alpha/beta diversity metrics, taxonomic alterations (phylum to species levels) in psoriasis cohorts), intervention and outcomes (dosage, duration of probiotic/FMT regimens), and biomarker correlations (e.g., serum inflammatory cytokines).

## 3. Results

### 3.1. Study Selection and Population Characteristics

**Figure 1:**
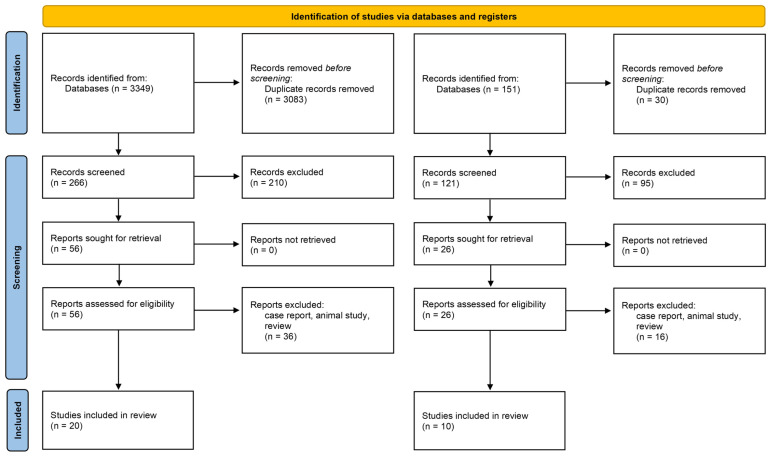
PRISMA flow diagram of study selection for inclusion in the systematic review.

Twenty studies investigating gut microbiota–psoriasis association met the final inclusion criteria, with detailed screening procedures delineated in Figure 1. A total of 266 studies were identified in the screening process, of which 56 underwent full-text review, and 20 were ultimately included for analysis (Table 1). All studies examined adults, and the psoriatic population was age- and sex-matched with the controls in all studies [8,9,10,11,12,13,14,15,16,17,18,19,20,21,22,23,24,25]. Plaque psoriasis cohorts were exclusively examined in seven studies [10,11,12,16,21,25,26], while six studies enrolled mixed psoriasis subtypes [8,9,14,17,18,19]. Psoriasis classifications remained unspecified in seven studies [13,15,27], and three studies stratified participants by disease severity (mild/moderate/severe) [16,17,28].

### 3.2. Analysis of α-Diversity in Psoriasis Studies

Alpha diversity was assessed using the following metrics: the Shannon index (diversity), Simpson index (evenness), and Chao1/ACE indices (richness). Substantial methodological heterogeneity across studies contributed to inconsistent findings. Six studies demonstrated decreased microbial diversity in psoriasis cohorts [8,13,14,15,18,25], five studies reported increased diversity [10,20,22,28,29], and eight studies showed no significant differences versus healthy controls [11,12,13,17,19,20,23] (Table 2). Notably, Dei-Cas [16] observed comparable diversity between psoriasis and non-psoriasis groups (*p* > 0.05), but identified diminished alpha diversity in moderate-to-severe psoriasis (ACE/Chao1 indices; mild vs. moderate-to-severe: *p* < 0.05).

### 3.3. Analysis of β-Diversity in Psoriasis Studies

Fourteen studies demonstrated distinct β-diversity patterns between psoriasis patients and healthy controls (Bray–Curtis/UniFrac metrics) [8,10,11,12,13,14,15,16,18,21,23,24,25,29] (Table 2). In contrast, three studies detected no significant β-diversity differentiation, potentially attributable to geographic or dietary confounding in cohort selection [20,22,28]. Interestingly, Wang et al. [23] demonstrated that microbial divergence was statistically significant only in subgroups with body mass index (BMI) < 25 kg/m^2^ (psoriasis vs. healthy: *p* < 0.05), but not in cohorts with BMI ≥ 25 kg/m^2^, suggesting obesity-mediated dysbiosis may override psoriasis-specific microbial alterations.

### 3.4. Taxonomic Alterations in Psoriasis Studies

**Figure 2:**
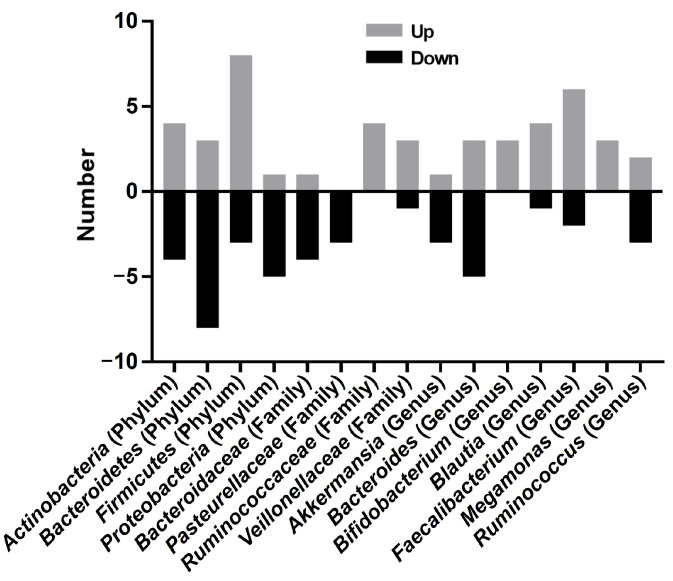
Taxonomic alterations in psoriasis studies.

Taxonomic profiling revealed marked heterogeneity in microbial composition (Figure 2) (Table 2). At the phylum level, *Bacteroidetes* and *Firmicutes* constituted the dominant phyla across both psoriasis patients and healthy controls. Most studies demonstrated elevated *Firmicutes* abundance and reduced *Bacteroidetes* levels in psoriasis cohorts versus healthy controls [8,11,13,15,16,19,20,30]. Contradictory evidence emerged from two independent cohorts: Huang et al. [14] and Wen et al. [28] documented increased *Bacteroidetes* and decreased *Firmicutes* levels in psoriasis patients. Similarly, *Actinobacteria* abundance exhibited opposed trends, being depleted in psoriasis (four studies reporting [8,14,19,30]) versus enriched (four studies [13,15,20,25]). The *Firmicutes*/*Bacteroidetes* (F/B) ratio, a putative biomarker of gut dysbiosis [11,13,15,16,17,20,30], was elevated in psoriasis cohorts, though inverted ratios were documented in Huang’s [14] and Wen’s [28] cohorts.

At the family taxonomic level, three studies described reduced *Bacteroidaceae* abundance in psoriasis cohorts compared to healthy controls [11,15,19,23]. In contrast, *Ruminococcaceae* exhibited elevated abundance in psoriasis patients [11,15,17,19]. Conflicting evidence emerged for *Lachnospiraceae*: two studies observed deletion [17,19], whereas three independent cohorts documented enrichment in psoriasis [11,15,23]. Notably, both *Ruminococcaceae* and *Lachnospiraceae* encompass key butyrate-producing taxa, suggesting functional implications for gut barrier integrity. *Veillonellaceae* abundance was consistently elevated across four studies [12,15,19,21], while *Enterococcaceae* showed marked abundance in psoriasis patients [12,19].

At the genus level, *Akkermansia* (belonging to the phylum *Verrucomicrobiota*, class *Verrucomicrobiae*, order *Verrucomicrobiales*, and family *Akkermansiaceae*) was increased in patients with psoriasis in one study [10], whereas a low abundance of *Akkermansia* was found in two studies [12,18]. Similarly, *Prevotella* (belonging to the phylum *Bacteroidetes*, class *Bacteroidia*, order *Bacteroidales*, and family *Prevotellaceae*) displayed reduced abundance in psoriasis patients compared to controls [13,20], while Zhao et al. and Zhang et al. documented increased levels in psoriasis cohorts [9,29]. *Faecalibacterium* (belonging to the phylum *Firmicutes*, class *Clostridia*, and order *Clostridiales*) showed enrichment in psoriasis patients [10,13,15,16,17,19,20], but Hidalgo et al. [15], Todberg et al. [25], and Wen et al. [28] reported contradictory depletion. *Blautia* (belonging to the phylum *Firmicutes*, class *Clostridia*, and order *Clostridiales*) demonstrated divergent patterns, being elevated in Dei-Cas et al.’s study [16], but reduced in Shapiro et al. [13], Hidalgo et al. [15], Todberg et al. [25], and Schade et al. [18]. *Ruminococcus* (belonging to the phylum *Firmicutes*, class *Clostridia*, order *Clostridiales*, and family *Molluscaceae*) abundance increased in psoriasis cohorts [13,15]. The abundance of *Bacteroides* was increased in two studies [12,14] and was decreased in four studies [15,16,29,31]. *Megamonas* (belonging to the phylum *Firmicutes*, class *Clostridia*, and order *Clostridiales*) showed elevated abundance [19,20,21], and *Ruminococcus* (belonging to the phylum *Firmicutes*, class *Clostridia*, order *Clostridiales*, and family *Peptococcaceae*) was increased in the studies by Shapiro et al. [13] and Hidalgo-Cantabrana et al. [15], whereas a decreased abundance was reported in the studies by Scher et al. [8] and Schade et al. [18].

### 3.5. Probiotics/FMT Intervention

Ten randomized trials investigated the efficacy of probiotics or FMT in modulating the gut microbiome and severity of psoriasis [26,32,33,34,35,36,37,38,39,40] (Table 3). The studies enrolled 604 psoriasis patients [33,34,37,38,39,40], with 89 concurrently diagnosed with PsA [26,36]. The intervention included *Bifidobacterium infantis* 35,624 (Groeger et al. [32]), *Lacticaseibacillus rhamnosus* (Suriano et al. [39]), Streptococcus salivarius K12 (Zangrilli et al. [40]), mix-strains (*Bifidobacterium longum* CECT 7347, *B. lactis* CECT 8145, and *Lacticaseibacillus rhamnosus* CECT 8361) (Navarro-López et al. [33]) (*Bifidobacterium* and *Lactobacilli*) (Siu et al. [38]) nine-strains (*Lacticaseibacillus* and *Bifidobacterium* by Haidmayer et al. [26], a multistrain probiotic including *Lactobacillus acidophilus*, *Bifidobacterium bifidum*, *Bifidobacterium lactis*, and *Bifidobacterium longum*) (Moludi et al. [34]), Lactocare^®^ Synbiotic (Akbarzadeh et al. [35]), probiotic capsules (Moludi et al. [37]), and FMT (Kragsnaes et al. [36]). The therapeutic duration ranged from 6 to 26 weeks. Probiotic interventions demonstrated significant improvements in quality of life and disease severity across psoriasis subtypes [32,33,34,37,38,40] and PsA [26]. However, FMT showed inferior efficacy versus a sham control in PsA [36].

**Table 2:** Characteristics of the included studies for investigating the gut microbiota–psoriasis.

No.	Author/Year/Ref	Method	Cases/Age/Female	α-Diversity (Ps vs. C)	β-Diversity (Ps vs. C)	F/B	Phylum (Ps vs. C)	Class (Ps vs. C)	Order (Ps vs. C)	Family (Ps vs. C)	Genus (Ps vs. C)	Species (Ps vs. C)
1	Scher et al., 2015 [8]	16s rRNA gene sequencing (V1-V2)	P (n = 15) 39.4 PsA (n = 16) C (n = 17) 42.2	Shannon index, Faith’s phylogenetic diversity index Lower diversity in psoriasis	Unweighted UniFrac analysis SD	NE	*Actinobacteria* ↓ *Firmicutes* ↑ *Bacteroidetes* ↓	*Actinobacteria* ↓	*Erysipelotrichales* ↓	*Erysipelotrichaceae* ↓ *Porphyromonadaceae* ↓	*Parabacteroides* ↓ *UC_Clostridia* ↓ *Coprobacillus* ↓ *Ruminococcus* ↓ *Akkermansia* ↓ *(PSA)* *Ruminococcus* ↓ *(PSA)*	*Coprococcus* species ↓
2	Eppinga et al., 2016 [9]	16s rRNA gene sequencing	P (n = 29) 46.0 ± 14.0 F (17) C (n = 33) 41 ± 14.9 F (23)	NE	NE	NE						*Escherichia coli* ↑ *F. prausnitzii* ↓
3	Doaa et al., 2016 [30]	16s rRNA gene sequencing	P (n = 45) 42.3 ± 10.0 C (n = 45) 44.2 ± 7.1	NE	NE	↑	*Actinobacteria* ↓ *Firmicute* ↑ *Bacteroidetes* ↓					
4	Codoñer et al., 2018 [10]	16s rRNA gene sequencing (V3-V4)	P (n = 52) C (n = 300) (from HMP)	Shannon Greater diversity in psoriasis	SD	NE					*Bacteroides* ↓ *Faecalibacterium* ↑ *Akkermansia* ↑	
5	Chen et al., 2018 [11]	16s rRNA gene sequencing (V3-V4)	P (n = 32) 42.8 ± 12.6 C (n = 64) 44.2 ± 10.8	Shannon index, Simpson index, Chao1 index NSD	UniFrac analysis (weighted and unweighted analyses), Bray–Curtis index SD (psoriasis patients with BMI < 25)	↑	*Bacteroidetes* ↓ *Firmicutes* ↑			*Bacteroidaceae* ↓ *Prevotellaceae* ↓ *Ruminococcaceae* ↑ *Lachnospiraceae* ↑		
6	Tan et al., 2018 [12]	16s rRNA gene sequencing (V4)	P (n = 14) 47.5 ± 4.7 C (n = 14) 40.4 ± 2.5	Shannon, Simpson, ACE, Chao1 NSD	PCA, UPGMA, SD	NE	*Verrucomicrobia* ↓ *Tenericutes* ↓	*Verrucomicrobiae* ↓ *Mollicutes* ↓	*Verrucomicrobiales* ↓	*S24-7* ↓ *Verrucomicrobiaceae* ↓ *Bacteroidaceae* ↑ *Enterococcaceae* ↑ *Veillonellaceae* ↑	*Akkermansia* ↓ *Bacteroides* ↑ *Enterococcus* ↑	*Akkermiansia muciniphila* ↓ *Clostridium citroniae* ↑
7	Shapiro et al., 2019 [13]	16s rRNA gene sequencing (V4)	P (n = 24) 52.7 ± 11.6 C (n = 22) 43.9 ± 12.7	Shannon, Chao1, Faith’s phylogenetic diversity index Lower diversity in psoriasis	UniFrac analysis (weighted and unweighted analyses) SD	↑	*Bacteroidetes* ↓ *Proteobacteria* ↓ *Firmicutes* ↑ *Actinobacteria* ↑				*Prevotella* ↓ *Lachnospira* ↓ *Faecalibacterium* ↑ *Ruminococcus* ↑ *Blautia* ↑ *Coprococcus* ↑ *Actinomyces* ↑ *Bifidobacterium* ↑ *Collinsella* ↑ *Dorea* ↑	*Ruminococcus gnavus* ↑ *Dorea formicigenerans* ↑ *Collinsella aerofaciens* ↑ *Prevotella copri* ↓
8	Huang et al., 2019 [14]	16s rRNA gene sequencing (V4–V5)	P (n = 16) 52.1 ± 3.0 C (n = 27) 52.9 ± 1.5	Shannon index, Simpson index—no difference ACE index and Chao1 index decreased in psoriasis	PCA based on the Bray–Curtis dissimilarity distance, SD	↓	*Firmicutes* ↓ *Proteobacteria* ↓ *Actinobacteria* ↓ *Bacteroidetes* ↑				*Carnobacterium* ↓ *Granulicatella* ↓ *Rothia* ↓ *Streptococcus* ↓ *Bacteroides* ↑ *Parabacteroides* ↑ *Lachnospira* ↑ *Lachnospiraceae*_UCG004 ↑ *Lactococcus* ↑ *Bacillus* ↑	
9	Hidalgo-Cantabrana et al., 2019 [15]	16s rRNA gene sequencing (V2–V3)	P (n = 19) 49 ± 11 C (n = 20) 43 ± 11	Shannon index, Chao1 index, Faith’s phylogenetic diversity index Lower diversity in psoriasis	Unweighted Unifrac analysis, SD	↑	*Bacteroidetes* ↓ *Proteobacteria* ↓ *Firmicutes* ↑ *Actinobacteria* ↑			*Bacteroidaceae* ↓ *Prevotellaceae* ↓ *Burkholderiaceae* ↓ *Lactobacillaceae* ↓ *Streptococcaceae* ↓ *Veillonellaceae* ↓ *Ruminococcaceae* ↑ *Lachnospiraceae* ↑ *Clostridiales Family* XIII ↑ *Bifidobacteriaceae* ↑ *Coriobacteriaceae* ↑	*Bacteroides* ↓ *Paraprevotella* ↓ *Barnesiella* ↓ *Parabacteroides* ↓ *Faecalibacterium* ↓ *Ruminococcus* ↑ *Blautia* ↑ *Bifidobacterium* ↑	
10	Dei-Cas et al., 2020 [16]	16s rRNA gene sequencing (V3–V4)	P (n = 55) 44.8 F (27) C (n = 27) 48.7 F (13)	Chao1 index NSD	SD	↑	*Bacteroidetes* ↓ *Firmicutes* ↑ *Proteobacteria* ↑ *Fusobacteria* ↑ *Verrucomicrobia* ↓				*Bacteroides* ↓ *Paraprevotella* ↓ *Faecalibacterium* ↑ *Blautia* ↑	
11	Yegorov et al., 2020 [17]	16s rRNA gene sequencing (V3–V4)	P (n = 14) 34.5 F (10) C (n = 7) 33.0 F (10)			↑	*Firmicutes* ↓			*Lachnospiraceae* ↓ *Ruminococcaceae* ↑	*Oscillibacter* ↓ *Roseburia* ↓ *Faecalibacterium* ↑	
12	Schade et al., 2021 [18]	16s rRNA gene sequencing (V3–V4)	P (n = 21) 50.1 ± 11.73 F (14) C (n = 24) 49.4 ± 10.06 F (15)	NE	NE	NE					*Ruminococcus* ↓ *Lachnospira* ↓ *Blautia* ↓ *Akkermansia muciniphila* ↓ *Dialister* ↑	*Akkermansia muciniphila* ↓ *Prevotella copri* ↑
13	Zhao et al., 2021 [29]	16s rRNA gene sequencing (V4)	P (n = 13) C (n = 13)	Observed species and Chao index of BT and N showed a significant difference. The ACE index of AT and N had a significant difference.		NE					*Bacteroides* ↓ *Clostridium* ↓ *Prevotella* ↑ *Lachnospira* ↑	
14	Zhang et al., 2021 [19]	16s rRNA gene sequencing (V3–V4)	P (n = 24) 43.13 ± 13.79 F (10) C (n = 30) 43.7 ± 13.21 F (10)	Sobs, Chao, ACE, Shannon, Simpson, Coverage NSD	SD	NE	*Bacteroidetes* ↓ *Firmicutes* ↑ *Actinobacteria* ↓	*Clostridia* ↑ *Fusobacteriia* ↓ *Actinobacteria* ↓	*Bacteriodales* ↓ *Bifidobacteriales* ↑ *Burkholderiales* ↓ *Aeromonadales* ↓ *Fusobacteriales* ↓	*Lachnospiraceae* ↓ *Veillonellaceae* ↑ *Ruminococcaceae* ↑ *Prevotellaceae* ↑ *Bacteroidaceae* ↓ *Enterobacteriaceae* ↑ *Fusobacteriaceae* ↓	*Faecalibacterium* ↑ *Megamonas* ↑ *Prevotella* ↑ *Bacteroides* ↓	*Prevotella_copri* ↑ *Faecalibacteriu*, *_prausnitzii* ↑ *Escherichia_coli* ↑ *Roseburia_faecis* ↓ *Bacteroides_uniformis* ↑
15	Xiao et al., 2021 [20]	Metagenomic sequencing	P (n = 30) 34, F 8 C (n = 15) 32, F 4	Shannon index was high	PCoA SD	↑	*Bacteroidetes* ↓ *Proteobacteria* ↓ *Euryarchaeota* ↓ *Actinobacteria* ↑ *Firmicutes* ↑ *Verrucomicrobia* ↑			*Oxalobacteraceae* ↓ *Porphyromonadaceae* ↓ *Pasteurellaceae* ↓ *Rikenellaceae* ↓ *Sphingobacteriaceae* ↓ *Comamonadaceae* ↓	*Prevotella* ↓ *Alistipes* ↓ *Eubacterium* ↓ *Butyricimonas* ↓ *Oxalobacter* ↓ *Actinobacillus* ↓ *Pseudoflavonifractor* ↓ *Faecalibacterium* ↑ *Bacteroides* ↑ *Bifidobacterium* ↑ *Megamonas* ↑ *Roseburia* ↑	*Faecalibacterium prausnitzii* ↑
16	Wang et al., 2021 [21]	16s rRNA gene sequencing (V4)	P (n = 20) C (n = 20)	Shannon, Simpson, Chao1, ACE	PCoA SD	NE		*Negativicutes* ↑ *Bacilli* ↑	*Lactobacillales* ↑ *Selenomonadales* ↑	*Veillonellaceae* ↑	*Romboutsia* ↓ *Megamonas* ↑	
17	Chang et al., 2022 [22]	Metagenomic sequencing	P (n = 33) 43.2 ±14.6 F (17) C (n = 15) 45.8 ±13.9 F (12)	Chao indices NSD Greater diversity in psoriasis (Shannon and Simpson indices)		NE						*Phascolarctobacterium succinatutens* ↓ *Bacteroides vulgatus* ↑ *Parasutterella excrementihominis* ↑
18	Wang et al., 2022 [23]	16s rRNA gene sequencing (V4)	P (n = 28) 44.5 F (9) C (n = 21) 46.1 F (6)	Shannon, Simpson, Chao1, ACE NSD	SD	NE	*Proteobacteria* ↓ *Bacteroidetes* ↑	*Clostridia* ↑ *Bacteroidia* ↓	*Clostridiales;* ↓ *Bacteroidales* ↑ *Enterobacteriales* ↓	*Enterobacteriaceae* ↓ *Peptostreptococcaceae* ↓ *Lactobacillaceae* ↑ *Muribaculaceae* ↑	*unidentified_Enterobacteriaceae* ↓ *unidentified_Lachnospiraceae* ↓ *Dorea* ↓ *Lactobacillus* ↑ *Dialister* ↑	*Escherichia_coli* ↓ *bacterium*_LF-3 ↓ *Parabacteroides_distasonis* ↑ *Bacteroides_thetaiotaomicron* ↑ *Lactobacillus_reuteri* ↑
19	Todberg et al., 2022 [25]	Metagenomic sequencing	P (n = 53) 48.0 F (24) C (n = 52) 49.0 F (23) Cohabitant partners (n = 21)	Shannon index NSD lower MGS richness in PP	SD	NE	*Actinobacteria* ↑ *Euryarchaeota* ↑			*Methanobacteriaceae* ↑	*Blautia* ↑ *Faecalibacerium* ↓	*Faecalibacterium sp. Ruminococcus torques* ↑ *Ruminococcus gnavus* ↑ F04-11AC ↓
20	Wen et al., 2023 [28]	Metagenomic sequencing	P (n = 32) C (n = 32)	Richness Shannon NSD	PCoA showed a minor separation	↓	*Firmicutes* ↓ *Bacteroidetes* ↑				*Roseburia* ↓ *Eubacterium* ↓	*Roseburia hominis* ↓ *Bacteroides eggerthii* ↓ *Bacteroides uniformis* ↑ *Escherichia* spp. ↑ *Alistipes finegoldii* ↑

Note: ↑ (Increased); ↓ (Decreased); Firmicutes/Bacteroidetes ratio (F/B); psoriatic arthritis (PsA); pustular psoriasis (PP); controls (C); not estimated (NE); no significant difference (NSD); significant difference (SD); human microbiome project (HMP); body mass index (BMI); before treatment (BT); after treatment (AT); metagenomic species (MGS); principal component analysis (PCA); unweighted pair group method with arithmetic mean (UPGMA); perform principal coordinates analysis (PCoA).

**Table 3:** Characteristics of studies included in the evaluation of probiotics or FMT in treating psoriatic disease.

No.	Author/Year/Ref	Study Subjects (n)	Intervention Group (n)/ Age/Female (n)	Control (n)	Intervention in the Study Group	Antipsoriasitic	Duration of Intervention	Study Type	Outcome Measurements	Microbiome and Biomarker	Conclusion (Supports the Hypothesis that Gut Microbiome Modulation via Ingestion Produces Clinical Improvement)
1	Groeger et al., 2013 [32]	Psoriasis patients (26)	n = 2	Placebo (14)	*Bifidobacteria infantis* 35,624		6–8 weeks	NE			Yes
2	Navarro-López et al., 2019 [33]	Psoriasis patients (90)	n = 46 41.57 ± 13.23, F (27)	Placebo (44) 43.09 ± 10.32 F (27)	*Bifidobacterium longum* CECT 7347, *B. lactis* CECT 8145, and *Lacticaseibacillus rhamnosus* CECT 8361	Topical corticosteroid betamethasone in combination with calcipotriol	12 weeks	randomized, double-blind, placebo-controlled trial	PASI, PGA recurrence ↓	genera Micromonospora and Rhodococcus disappearance Collinsella ↑ and Lactobacillus ↑	Yes
3	Haidmayer et al., 2020 [26]	PsA (58)	n = 58 F (7)	No control	Nine bacterial strains of *Lactobacillus* and *Bifidobacterium*	Anti-TNF, Anti-IL-17, Methotrexate, NSAID	12 weeks	pilot open-label study	mPASDAS ↓	fecal zonulin ↓, antitrypsin ↓, calprotectin ↓	Yes
4	Moludi et al., 2021 [34]	Psoriasis patients (50)	n = 25 42.70 ± 9.10 F (15)	Placebo (25) 43.10 ± 7.80, F (17)	Multistrain probiotics including *Lactobacillus acidophilus*, *Bifidobacterium bifidum*, *Bifidobacterium lactis*, and *Bifidobacterium longum*		8 weeks	single-center, randomized, placebo-controlled double-blind trial	BDI, PSS, PASI, and DLQI ↓TAC ↑, MDA ↓hs-CRP ↓, IL-6 ↓		Yes
5	Akbarzadeh et al., 2021 [35]	Psoriasis patients (36)	n = 22 F (9)	Placebo (14), F (7)	Lactocare^®^		12 weeks	double-blind, randomized, placebo-controlled study		serum levels of Fe, Zn, P, Mg, Ca, and Na are increased	Yes
6	Moludi et al., 2022 [37]	Psoriasis patients (46)	n = 23 42.04 ± 8.10, F (13)	Placebo (23) 43.76 ± 8.86, F (15)	Probiotic capsules (*Lactobacillus acidophilus*, *Bifidobacterium bifidum*, *Bifidobacterium lactis*, and *Bifidobacterium longum*)	Routine drug while taking any antioxidants was forbidden	8 weeks	randomized double-blind placebo-controlled clinical trial	QOL ↑hs-CRP ↓ IL1-β ↓, and LPS ↓		Yes
7	Suriano et al., 2023 [39]	Psoriasis patients (103)	n = 50 50 F (23)	Placebo (53) 52 F (27)	*Lacticaseibacillus rhamnosus*	Standard-of-care	6 M	a randomized, parallel, placebo-controlled, double-blind study	PASI, DLQI		No
8	Siu et al., 2024 [38]	Psoriasis patients (45)	n = 45 44.57 ± 11.5 F (17)	No control	*Bifidobacterium* and *Lactobacilli*	Usual medication or topical maintenance therapy	8 weeks	a single-arm, pre–post-interventional trial	PASI, BSFS ↓, DLQI ↑	a significant difference in the gut microbiome composition between the responders and non-responders	Yes
9	Zangrilli et al., 2022 [40]	Psoriasis vulgaris (198)	n = 100	Control (98)	*Streptococcus salivarius K12*	Topical treatments such as emollient and vitamin D derivatives	24 weeks	Prospective randomized controlled trial	PASI DLQI		Yes
10	Kragsnaes et al., 2021 [36]	PsA (31)	n = 15 42.04 ± 16.1 F (8)	Sham (16) 52.4 ± 11.0 F (12)	One gastroscopic-guided FMT or sham transplantation in combination with methotrexate	Intra-articular or systemic glucocorticoids and non-methotrexate conventional synthetic and biologic disease modifying antirheumatic drugs; a washout period of 12 weeks (26 weeks for biologic agents) was required	26 weeks		HAQ-DI, ACR20	FMT appeared to be inferior to sham in treating active peripheral PsA	No

Note: ↑ (Increased); ↓ (Decreased); Fecal microbiota transplantation (FMT); psoriatic arthritis (PsA); Psoriasis Area and Severity Index (PASI); Psoriasis Symptom Scale (PSS); Beck Depression Inventory (BDI); quality of life (QOL); Dermatology Life Quality Index (DLQI); total antioxidant capacity (TAC); high-sensitivity C-reactive protein (hs-CRP); lipopolysaccharides (LPS); Health Assessment Questionnaire Disability Index (HAQ-DI); American College of Rheumatology (ACR); Spanish Type Culture Collection (CECT); Physician Global Assessment (PGA); Modified Psoriatic Arthritis Disease Activity Score (mPASDAS); malondialdehyde (MDA); interleukin (IL); Dermatology Life Quality Index (DLQI); Lactocare^®^ contains seven strains (*Lacticaseibacillus casei*, *Lactobacillus acidophilus*, *Lacticaseibacillus rhamnosus*, *Lactobacillus bulgaricus*, *Bifidobacterium breve*, *Bifidobacterium longum*, and *Streptococcus thermophiles* with prebiotic fructooligosaccharide).

## 4. Discussion

The gut–skin axis represents an emerging framework elucidating bidirectional interaction between the microbiome and cutaneous pathophysiology through metabolites, immune mediators, and intestinal barrier integrity. Accumulating evidence indicates that normal intestinal microbiomes might modulate immune responses and protect the host against the development of inflammatory diseases. This review systemically evaluates intestinal microbiota profiles in patients with psoriasis versus healthy controls based on existing studies.

### 4.1. The Diversity of the Intestinal Microbiota in Psoriasis Patients Exhibited Marked Heterogeneity

The results demonstrated that the intestinal microbiota in patients with psoriasis presented a decreased diversity in six studies [8,13,14,15,18,25], increased diversity in five studies [10] [22,28,29], and no significant differences in eight studies, compared to that of healthy controls. Confounding factors such as regional disparities, unmatched controls (BMI, comorbidities), and incomplete medication histories likely contributed to inconsistent findings. Reduced α-diversity correlates with dysregulated immune–inflammatory pathways, particularly in interleukin-17/23 axis activation. Taxonomic shifts in Firmicutes/Bacteroidetes ratios may drive observed diversity discrepancies. The β-diversity analysis revealed distinct clustering between psoriasis and the healthy controls [8,10,11,12,13,14,15,16,18,21,23,24,25,29], indicating that the microbial communities differed between the two groups.

### 4.2. Firmicutes/Bacteroidetes Ratio and Metabolic Implications in Psoriasis

Psoriasis patients exhibit reduced *Bacteroidetes* abundance alongside elevated *Firmicutes* proportions, a pattern replicated across multiple studies [13,15,19,28]. *Firmicutes* dominance correlates with Gram-positive bacteria enrichment, whose peptidoglycan (PG) activates pro-inflammatory pathways via mutations in the (PGRP)-3 and PGRP-4 genes. Meanwhile, PG-specific T cells isolated from psoriatic skin lesions confirm PG-driven autoimmune responses [41]. Notably, Secukinumab et al. induced greater *Proteobacteria* enrichment and decreases in *Bacteroidetes* and *Firmicutes* [27].

The *Firmicutes/Bacteroidetes* (F/B) ratio is widely recognized as an important marker for assessing the state of the gut microbiota. In this review, most studies reported a significantly elevated F/B ratio in psoriasis patients [11,13,15,16,30]. This dysbiosis is associated with alterations in microbial metabolic output, particularly short-chain fatty acids (SCFAs), which play crucial immunomodulatory roles in immune-mediated diseases. Specifically, the observed increase in the F/B ratio correlates with elevated acetate production and reduced butyrate synthesis [42]. SCFAs exert immunomodulatory effects through multiple pathways. For instance, they promote the generation and function of regulatory T cells (Treg). Acetate modulates dendritic cell activity, influencing antigen presentation and immune response regulation [42]. Propionate stimulates intestinal epithelial cells to produce retinoic acids, a metabolite critical for immune tolerance and T-cell differentiation [43]. Butyrate directly enhances Foxp3 expression in T cells, a key transcription factor for Treg developments, thereby amplifying regulatory immune functions [44]. Through these mechanisms, SCFAs contribute to maintaining immune homeostasis and systemic metabolic balance by attenuating excessive inflammatory responses that are hallmarks of conditions such as inflammatory bowel disease, diabetes, cardiovascular disease, and obesity [45].

Moreover, butyrate is of particular physiological significance as the primary energy substrate for colonic epithelial cells. It regulates cellular proliferation and differentiation in the colonic mucosa, maintaining epithelial barrier integrity and renewal. Butyrate also exhibits potent anti-inflammatory, antioxidant, and anti-carcinogenic properties [42]. Reduced butyrate levels, associated with gut microbiota dysbiosis characterized by an elevated F/B ratio, may impair the mucosal layer barrier, thereby disrupting the gut epithelial barrier. This disruption can perpetuate chronic colonic and systemic inflammation, exacerbating inflammatory cascades in immune-mediated diseases [44]. Notably, clinical studies report conflicting findings on F/B ratio alterations in psoriasis. Doaa et al. [30] identified a positive correlation between the F/B ratio and the Psoriasis Area and Severity Index (PASI). Huang et al. [14] and Wen et al. [28] observed divergent trends in *Bacteroides* and *Firmicutes*. This discrepancy may stem from limited sample sizes and heterogeneous patient populations encompassing multiple psoriasis subtypes (plaque, pustular, erythrodermic, and PsA). Furthermore, an imbalanced F/B ratio has been linked to psoriasis-associated comorbidities, including cardiovascular diseases, obesity, insulin resistance, and nonalcoholic fatty liver disease [41].

### 4.3. Reduced Actinobacterial Phylum Abundance in Psoriasis and Anti-Inflammatory Effects of Bifidobacterium Supplementation

Multiple studies have demonstrated a significant reduction in the relative abundance of the *Actinobacteria* phylum in psoriasis patients [8,14,19,30]. Notably, PASI exhibited a statistically significant negative correlation with *Actinobacterial* phylum levels, suggesting a potential link between this microbial taxon and disease severity. This observation aligns with broader evidence implicating *Actinobacteria* in modulating inflammatory and immune-related pathologies. Oral administration of *Bifidobacterium* spp. (*Actinobacteria* phylum) has shown therapeutic promise in psoriasis. Studies indicate that probiotic *Bifidobacterium* supplementation reduces intestinal inflammation and mitigates systemic autoimmune responses by suppressing pro-inflammatory bacteria taxa [26,32,33,34,35,38,39,40]. Furthermore, fecal *Bifidobacterium* levels inversely correlate with inflammatory biomarkers, highlighting its immunoregulatory role.

Groeger et al. [32] investigated the effects of *B. infantis* 35,624 supplementation (6–8 weeks) in psoriasis. Treatment significantly reduced plasma reactive protein (CRP) levels compared to placebo. Additionally, lipopolysaccharide (LPS)-stimulated TNF-α and IL-6 secretion by peripheral blood mononuclear cells (PBMCs) was attenuated, demonstrating systemic anti-inflammatory effects. Navarro-López et al. [33] conducted a randomized controlled trial using a probiotic mixture (*B. longum* CECT 7347, *B. lactis* CECT 8145, and *Lacticaseibacillus rhamnosus* CECT 8361). The probiotic group exhibited greater reductions in PASI score and a lower relapse rate (20% vs. 41.9% in placebo) over 12 weeks. Gut microbiota analysis revealed *Collinsella* and *Lactobacillus* enrichment alongside *Micromonospora* and *Rhodococcus* depletion, suggesting strain-specific modulation. Akbarzadeh et al. [35] reported increased serum mineral levels (Fe, Zn, P, Mg, Ca, and Na) in psoriasis patients receiving the seven-strain probiotic Lactocare^®^ (*Lacticaseibacillus casei*, *L. acidophilus*, *L.rhamnosus*, *L.bulgaricus*, *Bifidobacterium breve*, *B. longum*, and *Streptococcus thermophiles* with prebiotic fructooligosaccharide), indicating improved nutrient absorption. Moludi et al. [37] observed reduced serum lipopolysaccharide (LPS), hs-CRP, and IL1-β levels after 12 weeks of probiotic therapy (*Lactobacillus acidophilus*, *Bifidobacterium bifidum*, *B. lactis*, and *B. longum*), further corroborating anti-inflammatory mechanisms.

Probiotic supplementation demonstrated clinically meaningful reductions in psoriasis severity and inflammatory biomarkers. However, current evidence is limited to heterogeneous study designs (e.g., varying strains and dosages), small sample sizes, and mixed psoriasis subtypes (plaque, erythrodermic, PsA), and lack of mechanistic data linking microbiota shifts to immune pathways. Therefore, large-scale multicenter RCTs and longitudinal studies are needed to standardize probiotic formulations and dosing regimens, elucidate microbiota–host interactions through multi-omics approaches, and evaluate long-term efficacy in psoriasis.

### 4.4. Elevated Ruminococcaceae Family Abundance in Psoriasis and Its Functional Implications

Multiple studies report a significantly increased abundance of the *Ruminococcaceae* family in psoriasis patients compared to healthy controls [5,15,17,19]. This dysbiosis likely arises from immune–inflammatory crosstalk in psoriasis, where T helper 17 (th17)-mediated inflammation may promote a gut microenvironment favoring *Ruminococcaceae* proliferation. However, the precise molecular mechanisms driving this shift require further investigation. Conversely, reduced *Lachnospiraceae* family abundance has been consistently observed [17,19]. As a key regulator of gut homeostasis, *Lachnospiraceae* contribute to bile acid metabolism and secondary bile acid synthesis, SCFA production (particularly butyrate), and immune tolerance through dendritic cells. Both *Ruminococcaceae* and *Lachnospiraceae* are primary butyrate producers. Butyrate serves as an important energy source for colonocytes, helps in maintaining the integrity of the intestinal barrier, and also has anti-inflammatory properties [44]. Therefore, the changes in the abundances of *Ruminococcaceae* and *Lachnospiraceae* could potentially impact the production of butyrate and subsequently influence gut health and the progression of psoriasis.

### 4.5. The Abundance of Prevotella and Bacteroides Genera Was Decreased, and Megamonas, Ruminococcus, and Faecalibacterium Were Increased in Psoriasis

The abundance of *Prevotella* was significantly reduced in psoriasis patients across four studies. The genus is associated with high-fiber diets and predominates in non-westernized populations. Haidmayer et al. [26] carried out a pilot study and detected evidence of increased gut permeability and inflammation in PsA patients. When the PsA patients were administered these probiotic strains, it led to a welcome decrease in intestinal permeability. Even more encouragingly, the amelioration of disease activity became evident. However, the effects were not long-lasting following the termination of the treatment.

*Megamonas* was significantly elevated in psoriasis patients, correlating with pro-inflammatory cytokines (TNF-α, IL-6) [19,21]. A study by Wang et al. [21] has shown that *Megamonas* is a pro-inflammatory bacterium, is significantly associated with systemic inflammatory cytokines, and may be involved in inflammation in metabolic diseases and mental illnesses. *Megamonas* was recognized as a biomarker for T2DM when evaluating the association between type 2 diabetes and the gut microbiota [21]. In addition, a study of childhood obesity in Mexico found that the number of *Megamonas* was twice as high as in non-obese children [46].

The abundance of *Ruminococcus* was reported to be increased according to Shapiro et al. [13] and Hidalgo-Cantabrana et al. [15]. Conversely, Scher et al. [8] and Shade et al. [18] identified a lower abundance of the genus *Ruminococcus* in patients with PsA. Such discrepancies in the abundance of this particular genus across different studies highlight the complexity of the gut microbiota and its relationship with these autoimmune conditions. *Ruminococcus* species play a significant role in maintaining gut health, as they are mucin-degrading. Mucins are glycoproteins that form an important protective layer on the surface of the intestinal epithelium. By degrading mucins, *Ruminococcus* species participate in the recycling of nutrients and the modification of the gut environment. This regulation is particularly achieved through the production of SCFAs.

Four independent cohorts demonstrated a significant reduction in *Bacteroides* abundance in psoriasis patients compared to healthy controls [15,16,29,31]. *Bacteroides* play a key role in intestinal homeostasis, which can produce SCFAs to induce the production of IL-10 in the colon and increase the number of Treg cells in the mucosa. SCFA-producing microbiota and SCFAs are effective regulators of T cells. Experiments have shown that gut flora can affect the differentiation of primitive T cells, and the differentiated Treg cells, which are potential pathogens and usually act as symbionts in healthy individuals, can inhibit Th17 cells from attacking pathogens [42].

The abundance of *Faecalibacterium* was increased in psoriasis patients compared with the control population [10,13,15,16,17,19]. However, a study found that stool samples from psoriasis patients were depleted of *Faecalibacterium prausnitzii* based on a PCR-aided identification of specific bacterial species in the Netherlands. Notably, gut *Faecalibacterium* alterations have been associated with eczema and IBD, and, taken together, these data highlight the dynamic role gut *Faecalibacterium* spp. are potentially playing in the pathogenesis of both skin and gut diseases [9]. Eppinga et al. [31] found that psoriasis patients harbored a significantly lower abundance of anti-inflammatory bacterium *F. prausnitzii* species in their stool and a significantly higher abundance of *Escherichia coli* than healthy controls, which was similar to IBD patients [47]. The study demonstrates an IBD-like decrease in *F. prausnitzii* together with an increase in *E. coli* in psoriasis, supporting the presence of a gut–microbiome–skin axis in psoriasis and IBD [48].

### 4.6. Limited Evidence for Fecal Microbiota Transplantation in Psoriasis, Requiring Further Validation

Despite emerging interest in FMT as a microbiome-targeted therapy for psoriasis, clinical evidence remains scarce. The phase II randomized double-blind trial by Kragsnaes et al. [36] revealed critical insights. FMT showed inferior efficacy versus a sham control in PsA; there was greater reduction in the Health Assessment Questionnaire Disability Index (HAQ-DI) with the sham (*p* = 0.03). There was no intergroup difference in the American College of Rheumatology (ACR20) 20% improvement response rate (32% FMT vs. 36% sham; *p* = 0.72). There were comparable adverse events between groups (diarrhea: 28% FMT vs. 21% sham). More studies are essential to accurately assess the role of FMT in psoriasis, not only to understand its potential benefits but also to identify any potential risks or adverse effects that might have been overlooked in this initial evaluation.

## 5. Conclusions

This systematic analysis reveals that the gut microbiota of psoriasis patients differs from that of healthy controls, though the results exhibit substantial heterogeneity. While studies reported an increased abundance of *Firmicutes* and *Ruminococcaceae*, alongside decreased *Actinobacteria* and *Bifidobacterium*, these findings were inconsistently replicated across cohorts. Importantly, multiple confounding factors, including patient demographics, BMI, lifestyle variations, geographic diet patterns, and comorbidities, may account for observed microbiota disparities. This underscores the complexity of the gut microbiota–psoriasis relationship and highlights the need for standardized methodologies in future investigations. Notably, probiotic intervention demonstrated a state associated with reduced psoriasis severity (e.g., PASI score improvement). This finding not only supports the potential of microbe-targeted therapy in psoriasis management but also warrants future research to elucidate the causal mechanism. Future studies should prioritize longitudinal design, strain-specific probiotic analyses, and integration with host immune profiling to develop precision microbiome modulation strategies.

## Figures and Tables

**Table 1:** Inclusion/exclusion criteria.

Gut microbiota–psoriasis investigation	Keywords	“gastrointestinal microbiome”, “gut microbiota”, “intestinal microbiome”, “intestinal microbiota” “bacteria”, “dysbiosis”, “gut”, “gastrointestinal”, “intestine”, “stool”, “fecal”, and “psoriasis”
	Inclusion criteria	Human case–control studies investigating the association between the gut microbiota and psoriasis; Usage of culture-independent, high-throughput sequencing methods for gut microbiota quantification; Articles published in English.
	Exclusion criteria	Review papers, conference abstracts, case reports, expert opinions, editorials, and studies using animal models
Probiotics or FMT in treating psoriasis	Keywords	“Probiotics” or “Fecal microbiota transplantation” and “psoriasis”.
	Inclusion criteria	Human case–control studies investigating the efficacy of probiotics or fecal microbiota transplantation in psoriasis
	Exclusion criteria	1. Review papers, conference abstracts, case reports, expert opinions, editorials, and studies using animal models; 2. Patients were ineligible if they had comorbidities—inflammatory bowel diseases (Crohn’s disease, ulcerative colitis), rheumatoid arthritis, ankylosing spondylitis, onset of a severe organ dysfunction, terminal illness, human immunodeficiency virus (HIV) infection, or cancer—throughout the study duration; patients treated with antibiotics during the last 8 weeks.

## Data Availability

Not applicable.
